# Comprehensive macro and micro views on immune cells in ischemic heart disease

**DOI:** 10.1111/cpr.13725

**Published:** 2024-08-01

**Authors:** Yongjian Zhao, Mingyue Tan, Yunfei Yin, Jun Zhang, Yiyi Song, Hang Li, Lin Yan, Yifeng Jin, Ziyue Wu, Tianke Yang, Tingbo Jiang, Hongxia Li

**Affiliations:** ^1^ Department of Cardiology The First Affiliated Hospital of Soochow University Suzhou Jiangsu China; ^2^ Department of Geriatrics, Southwest Hospital The Third Military Medical University (Army Medical University) Chongqing China; ^3^ Suzhou Medical College of Soochow University Jiangsu China; ^4^ Department of Ophthalmology, The First Affiliated Hospital of USTC University of Science and Technology of China Hefei China

## Abstract

Ischemic heart disease (IHD) is a prevalent cardiovascular condition that remains the primary cause of death due to its adverse ventricular remodelling and pathological changes in end‐stage heart failure. As a complex pathologic condition, it involves intricate regulatory processes at the cellular and molecular levels. The immune system and cardiovascular system are closely interconnected, with immune cells playing a crucial role in maintaining cardiac health and influencing disease progression. Consequently, alterations in the cardiac microenvironment are influenced and controlled by various immune cells, such as macrophages, neutrophils, dendritic cells, eosinophils, and T‐lymphocytes, along with the cytokines they produce. Furthermore, studies have revealed that Gata6^+^ pericardial cavity macrophages play a key role in regulating immune cell migration and subsequent myocardial tissue repair post IHD onset. This review outlines the role of immune cells in orchestrating inflammatory responses and facilitating myocardial repair following IHD, considering both macro and micro views. It also discusses innovative immune cell‐based therapeutic strategies, offering new insights for further research on the pathophysiology of ischemic heart disease and immune cell‐targeted therapy for IHD.

## Graphical abstract

This review describes the mechanisms of immune cell‐mediated inflammatory responses in the course of ischemic heart disease from macroscopic and microscopic perspectives, explains the mechanisms of macrophage‐induced activation of fibroblasts during the repair phase, and describes emerging therapeutic options derived from the immune system.

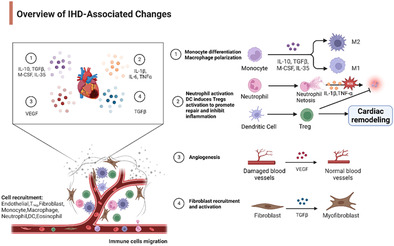

## 1 INTRODUCTION

Ischemic heart disease (IHD) is defined by diminished blood flow to the heart muscle, creating an imbalance between the oxygen supply and demand of the myocardium. Ischemic heart disease, which is prevalent globally, often stems from atherosclerotic blockages or obstructions in the coronary arteries due to factors such as arterial spasm or microvascular dysfunction.^1^ In more than 90% of cases, the root cause is excessive lipid accumulation within the coronary arteries. This accumulation can lead to the narrowing or complete obstruction of these vessels, resulting in the formation of atherosclerotic plaques along the arterial walls and a consequent reduction in blood flow to the heart muscle.^2^, ^3^

The cardiovascular system closely interacts with the immune system in organisms. The innate immune system, with its rapid response to pathogens and stressors, is a critical defence line against injuries.^3^ Monocytes in the bloodstream and resident immune cells within tissues are key players in preserving the equilibrium of cardiovascular health. Cardiovascular diseases (CVDs) impact not only the cardiovascular system but also the immune, lymphopoietic, and nervous systems to different extents, with the most significant effects observed on the immune system.^4^ Clinical evidence underscores the vital influence of the innate immune system on the prognosis of individuals with IHD. The activation of this natural defence mechanism and the subsequent inflammatory response are central to the development of IHD. Unchecked and excessive inflammation can hasten the progression of adverse ventricular remodelling and fibrosis, highlighting the regulation of immune system‐driven inflammation as a crucial therapeutic avenue for enhancing outcomes in IHD patients.^3^ The cardiac remodelling process after acute myocardial infarction includes recruiting immune cells, forming the infarct area, absorbing necrotic tissue, phagocytosis, activating myofibroblasts, scarring, and angiogenesis.^5^, ^6^ Increasing evidence on inflammation's role in IHD highlights the importance of understanding the immune system's involvement, particularly immune cells, in its development.

In this review, we delve into the nuanced alterations in the immune cell‐driven inflammatory response during the development of IHD by examining both macroscopic and microscopic perspectives. From a macro perspective, immune cells, whether residing in or infiltrating cardiac tissue, are pivotal for sustaining cardiac balance and facilitating tissue repair.^7^, ^8^ In the aftermath of IHD leading to acute myocardial infarction (AMI), a surge of activated immune cells orchestrates the inflammatory response and aids in myocardial tissue recovery. This includes the mobilization and activation of monocyte macrophages,^9^ the early release of pro‐inflammatory agents by neutrophils,^10^ the involvement of dendritic cells (DCs) and regulatory T cells (Tregs) in tissue repair, the participation of eosinophils (EOS) in the inflammatory process^11^ and the activation of fibroblasts during myocardial repair, all of which play vital roles.^12^

From a micro perspective, the inflammatory response post‐IHD involves immune cells engaging in distinct roles. Upon activation, monocyte macrophages differentiate into either pro‐inflammatory M1 macrophages, which emit cytokines such as tumour necrosis factor (TNF)‐α and interleukin (IL)‐1β, or anti‐inflammatory M2 macrophages, which release cytokines such as IL‐10 and transforming growth factor (TGF)‐β, which cater to the early and later stages of inflammation, respectively,^13^ and the polarization of macrophages is also regulated by their own metabolism and various cytokines.^9^ In addition, the lack of a special type of macrophage—Gata6 ^+^ pericardial cavity macrophage (GPCM) has a harmful effect on the process of fibrosis repair after myocardial infarction^14^; as the earliest cells to accumulate in the damaged area after AMI, neutrophils are critical for initiating and activating inflammation after infarction. In cases of local injury or infection, neutrophils rapidly accumulate and adhere to activated endothelial cells, after which they translocate to tissues to phagocytose pathogens via their unique neutrophilic extracellular trapping network (NETosis) and produce antimicrobial proteins and reactive oxygen species (ROS) to phagocytose and eliminate necrotic cell debris.^15^, ^16^ EOSs are often considered critical for mediating inflammatory stimulation. Nevertheless, an in‐depth study of the immune response after IHD revealed the opposite effect of EOS.^17^ We believe that the functions of EOS will gradually be discovered. In addition, this article discusses the mechanism by which macrophages activate fibroblasts during myocardial repair and summarizes new IHD immunotherapies, including chimeric antigen receptor (CAR)‐T‐cell therapy. We believe that fibroblast activation proteins can be potential therapeutic targets for regulating immune cell activation and inducing fibrosis. Generally, the use of immune cells to regulate the inflammatory response in IHD patients represents a promising treatment approach and may provide new insights and methods for enhancing clinical outcomes after IHD.

## 2 COMPREHENSIVE OVERVIEW OF THE IMMUNE RESPONSE IN IHD

**FIGURE 1:**
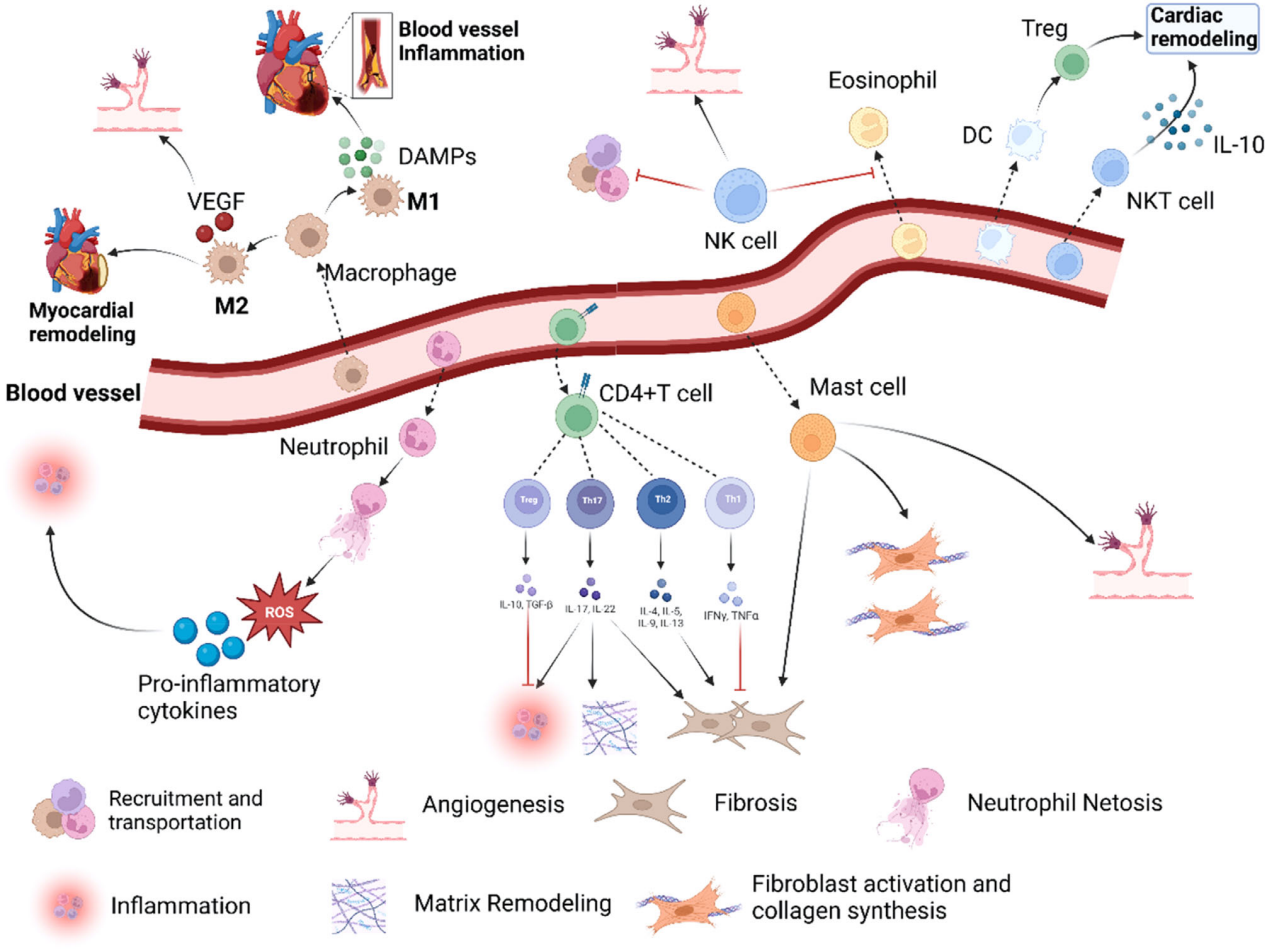
Immune cells in the cardiovascular system. After AMI, monocytes are recruited to cardiac tissue and differentiate into M1 and M2 macrophages. M1 macrophages induce vascular inflammation by releasing DAMPs, and M2 macrophages promote damaged tissue repair by producing VEGF, among others. Pro‐inflammatory cytokines induce neutrophil infiltration, and neutrophils mediate the release of ROS through neutrophil NETosis and secrete inflammatory cytokines that exacerbate and sustain the inflammatory response. Infiltrating CD4^+^ cells include helper T cells (Th1 and Th1 cells reduce the fibrotic response, while Th2 and Th17 cells promote fibrosis. Th17 cells also promote inflammation and extracellular matrix remodelling, while Treg cells attenuate the inflammatory response). Mast cells promote fibrosis, and angiogenesis and promote fibroblast proliferation and collagen synthesis via TNF‐α. NK cells reduce cardiac eosinophil infiltration and prevent the maturation and transport of inflammatory cells. DCs induce myocardial hypertrophy and improve ventricular remodelling by mediating the increase in Treg activity. The secretion of cytokines such as IL‐10 by NKT cells plays a role in attenuating the inflammatory response and adjusting ventricular remodelling.

Massive cardiomyocyte death, activation of the innate immune system, and widespread inflammation are common pathological features of IHD. These pathological features contribute to the progression of IHD and may lead to further complications if not properly managed.^18^ The acute inflammatory response after IHD is a critical factor in determining the final area of myocardial infarction and adverse ventricular remodelling, rendering the regulation of the inflammatory response an essential target for cardiac protection.^19^ During the innate immune response, activated immune cells aggregate in the ischemic myocardium to coordinate the initial inflammatory response, which is primarily aimed to clear dead cell debris in the infarct area and prepare for the subsequent repair response. Within the first few hours after IHD, neutrophils predominantly infiltrate the infarct area, triggering an immune cell response, and the accumulation of pro‐inflammatory monocytes and macrophages in the infarct area after 48 h leads to the formation of an apparent pro‐inflammatory environment, which results in cardiomyocyte death and myocardial tissue injury.^19^ On the 4th to 7th days after IHD, anti‐inflammatory monocytes and macrophages are activated to mediate changes in the inflammatory repair phase by inhibiting and suppressing the initial inflammatory response, thereby promoting wound healing and scar repair.^20^ Therefore, a sustained or prolonged inflammatory phase response can exacerbate myocardial injury, leading to enlarged infarct size and excessive cardiac remodelling.^3^, ^21^ Moreover, some typical immune cells, including T lymphocytes, DCs, and EOSs, also play crucial roles in the attenuation of inflammation and the repair of myocardial tissue fibrosis (Figure 1). The inflammatory cascade after AMI plays a crucial role in cardiac injury, with cytokines released by immune cells being extensively involved in the cardiac inflammatory response after IHD and subsequent repair processes. Despite recent questions about the role of inflammatory signalling in ischemic injury, treatments targeting the innate immune response may undeniably provide a promising therapeutic strategy to limit infarct size, improve adverse remodelling, and enhance cardiac function. Consequently, we will concentrate on these immune cells from both macro and micro perspectives in the following sections.

## 3 MONOCYTES AND MACROPHAGES IN IHD

### 3.1 Mechanism of monocyte and macrophage activation and initiation in IHD

The monocyte–macrophage system consists of myeloid cells, such as monocytes, macrophages, and DCs. Monocytes arise from bone marrow and extramedullary haematopoietic sites (e.g., the spleen). Upon infiltration into damaged tissue, monocytes participate in the inflammatory response by either directly producing TNF‐α and nitric oxide (NO) or indirectly differentiating into macrophages and DCs.^22^, ^23^

Monocytes/macrophages represent the earliest immune cell type responding to ischemic injury.^24^ After AMI, cardiac macrophages quickly diminish; hence, post‐myocardial infarction macrophage recruitment is thought to primarily involve monocytes originating from haematopoietic areas. Alongside neutrophils, these cells are attracted to the infarct site promptly during infarction to trigger a robust inflammatory reaction to tissue damage.^25^, ^26^

Macrophages, one of the largest groups of immune cells, are involved in innate and adaptive immunity and play important roles in the removal of pathogens, tissue inflammatory damage and fibrosis repair. Macrophages are key effectors of immune cell responses to tissue damage and are emerging as therapeutic targets for inflammation‐related diseases, including ischemic heart disease and atherosclerosis. When IHD occurs, macrophages have both pro‐inflammatory and anti‐inflammatory effects.^27^, ^28^, ^29^ Injury cardiomyocytes and various immune cells release cytokines to polarize macrophages. There are two types of polarized macrophages: classically activated macrophages (M1 macrophages) and alternatively activated macrophages (M2 macrophages). M1 macrophages express high levels of pro‐inflammatory cytokines such as TNF‐α, IL‐6, and cytotoxic (NO, ROS) mediators that activate other immune cells and induce microbicidal activity, leading to the clearance of microbial infections and promoting the formation of a pro‐inflammatory environment.^30^, ^31^ In contrast, M2 macrophages can downregulate inflammatory mediators, release anti‐inflammatory cytokines (IL‐4 and IL‐10) and the mannose receptor (MR), and promote the expression of various tissue repair factors, which participate in tissue repair and immunomodulation.^13^, ^32^ The balance between M1 and M2 macrophages plays a vital role in the healing and remodelling of the damaged myocardium.

Investigations on macrophage phenotypes revealed that macrophages activated by pro‐inflammatory signals (e.g., lipopolysaccharide or LPS and interferon (IFN)‐γ) exhibited a specific pro‐inflammatory transcription profile. Activated M1 macrophages enhanced cytophagy, MHC‐II‐related antigen presentation, and ROS production. They also produce and release pro‐inflammatory cytokines (e.g., IL‐12, IL‐23, and TNF‐α), chemokines (CXCL1, CXCL2, and CXCL5), matrix metalloproteinases (MMP‐1, 2, 7, 9, 12) and inducible nitric oxide synthase (iNOS), which lead to the production of large amounts of NO and help eliminate pathogens. These inflammatory mediators form a pro‐inflammatory microenvironment that lays the foundation for subsequent immune cell action.^13^, ^22^

**FIGURE 2:**
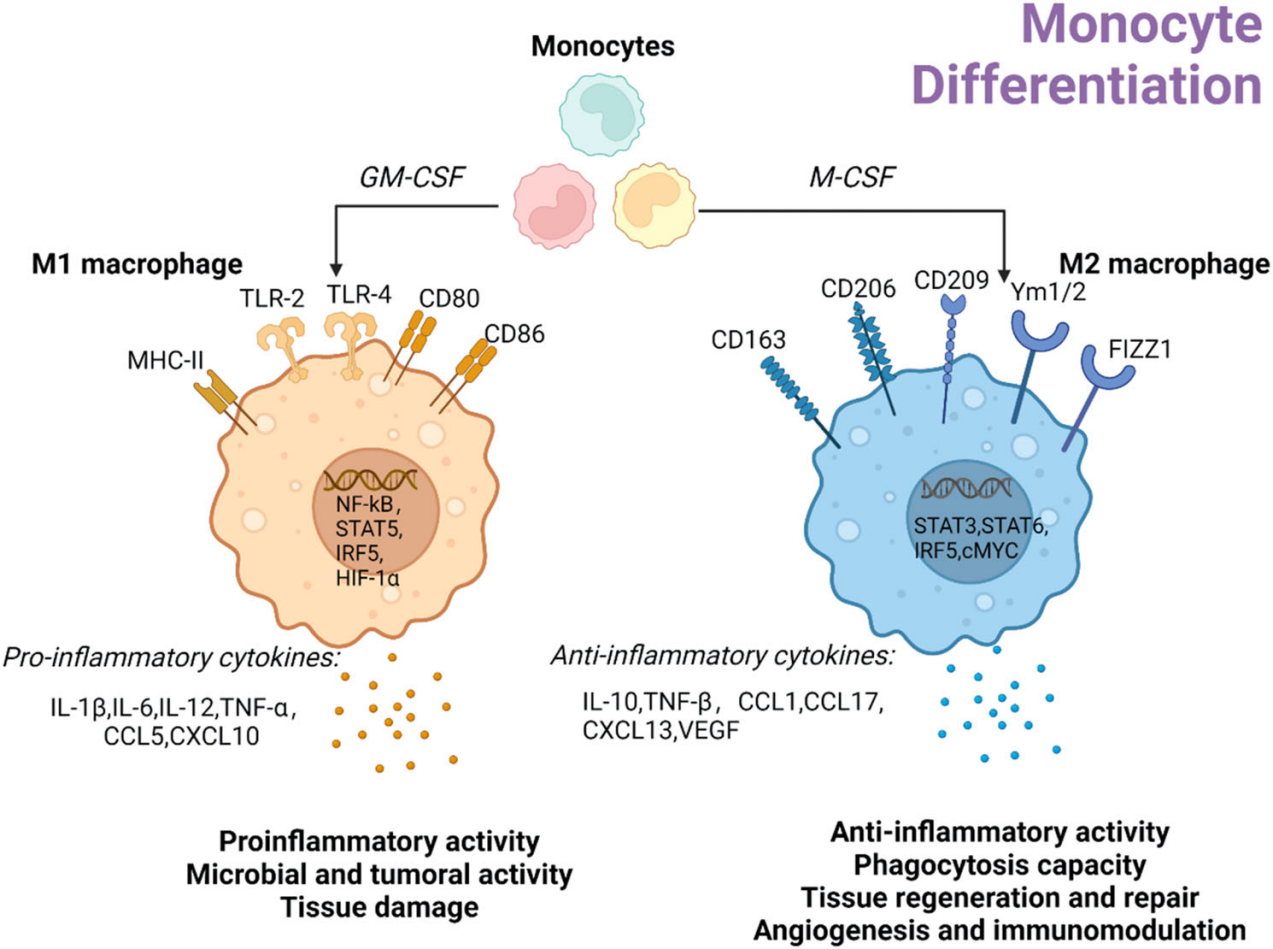
Monocyte differentiation after AMI. During IHD, bone marrow‐derived monocytes are recruited to the damaged myocardial tissue and subsequently differentiate to form M1 macrophages and M2 macrophages stimulated by GM‐CSF and M‐CSF. M1 macrophages possess markers such as TLR‐2, TLR‐4, CD80, and CD86 and produce pro‐inflammatory cytokines including IL‐1β, TNF‐α, and CCL5, which induce M2 macrophages to express markers such as CD163, CD206, and FIZZ1, and produce anti‐inflammatory cytokines including IL‐10, TNF‐β, and VEGF, which induce tissue repair.

In contrast, stimulation of macrophages with anti‐inflammatory cytokines (e.g., IL‐4 and IL‐13) led to the expression of anti‐inflammatory genes and transformation of the repair phenotype.^22^ Activated M2 macrophages produce anti‐inflammatory cytokines (IL‐10), chemokines (CCL17), and growth factors (VEGF and TGF‐β) (Figure 2). These mediators stimulate fibroblast activation, induce extracellular matrix production, angiogenesis, and cell proliferation, and promote tissue repair and remodelling.^22^, ^33^, ^34^

**FIGURE 3:**
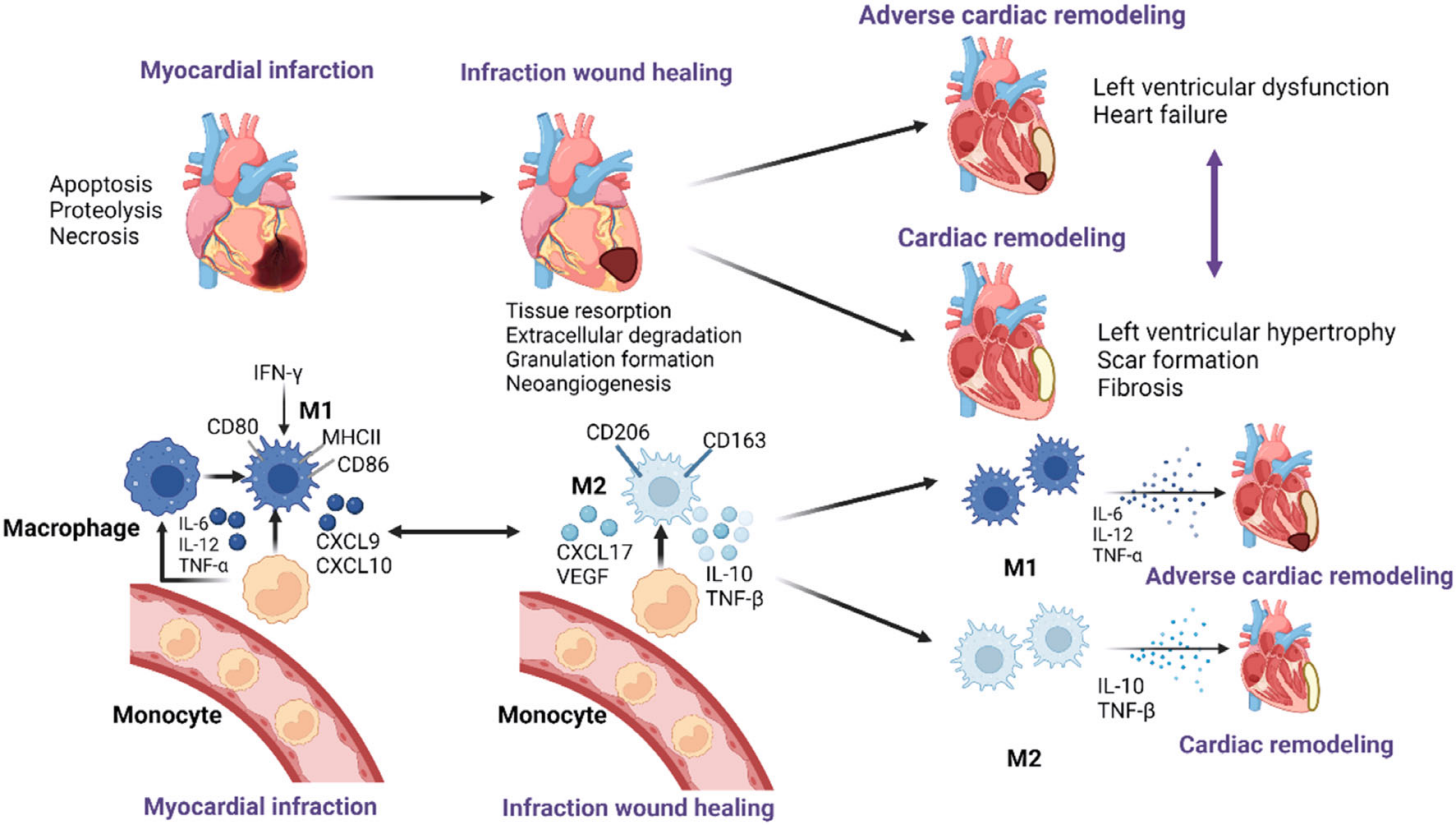
Macrophage‐mediated‐remodelling of two opposing ventricles after infarction. After AMI caused by multiple factors, myocardial cells die in large numbers and inflammatory cells infiltrate into the damaged tissue to mediate the repair response after injury. Monocytes recruited from the bone marrow differentiate in the damaged tissue to form macrophages, which subsequently differentiate into pro‐inflammatory M1 macrophages and anti‐inflammatory M2 macrophages. In the post‐infarction repair phase, if M1 macrophages persist, they lead to long‐term maintenance of the inflammatory response, which in turn leads to adverse ventricular remodelling and eventually heart failure; if M1 macrophages are able to dissipate, M2 macrophages play a key role in the repair phase, producing anti‐inflammatory and repair mediators that induce benign ventricular remodelling.

Macrophages are dynamic immune cells with various functions and play a key role in cardiovascular diseases. In ischemic heart disease, white blood cells invade the heart, triggering inflammation. Reduced macrophage numbers can hinder heart healing and worsen outcomes. Notably, anti‐inflammatory M2 macrophages are essential for inducing infarct healing.^29^, ^35^ The pro‐inflammatory and anti‐inflammatory phases are critical to the success of myocardial repair after acute myocardial infarction. At the beginning of the inflammatory phase, M1 macrophages predominate, producing inflammatory cytokines, removing necrotic cellular debris from damaged tissues and inhibiting infectious agents, laying the groundwork for the repair phase to regulate infarct size and left ventricular remodelling.^36^, ^37^ In the late inflammatory phase, pro‐inflammatory M1 macrophages are transformed into anti‐inflammatory M2 macrophages, and M2 macrophages begin to dominate, thereby protecting the heart from the adverse effects of acute myocardial infarction.^38^ Macrophages respond to strain by promoting the expression of inflammatory factors and scavenger receptors, which protect the damaged myocardium and promote tissue repair (Figure 3).^24^, ^39^

Following an episode of IHD, it is crucial to shift the balance between M1 and M2 macrophages toward M2 to the greatest extent possible. This strategic alteration is essential for promoting cardiac repair and enhancing myocardial function after injury.^22^, ^40^, ^41^ Further exploration of the specific functions of macrophage subtypes in the context of IHD could enhance our knowledge of the various responses to myocardial injury. It also sets the stage for targeted interventions focusing on particular subtypes. This method is on the verge of revealing effective therapeutic approaches for future advancements.^42^

### 3.2 Chemokines improve the cardiac inflammatory response by regulating macrophages in IHD

Chemokines, as inflammatory mediators, play a key role in the immune response, immune injury, and coordination of immune cell recruitment to sites of inflammatory injury.^43^ Notably, chemokines such as CXCL8 mediate neutrophil recruitment, and CCL2 and CXCL1 are known to be potent regulators of monocytes, T cells, and neutrophils.^44^

CXCL1, CXCL2, and CXCL5 share a common receptor, CXCR2, which is expressed on neutrophils, monocytes, macrophages, and mast cells and is involved in the transport of these leukocytes under homeostatic conditions and during inflammation.^45^ In addition to their roles in immune cell recruitment and cellular homeostasis, there is growing evidence for a relationship between multiple CXC chemokines and the development of CVD. CXCL1 and CXCL2 regulate the activation of macrophage inflammatory vesicles, which influences macrophages to play a pivotal role in the inflammatory response after IHD.^43^, ^46^

Chemokines play a central role in many key processes of the inflammatory response related to cardiovascular events, including the early stage of monocyte differentiation, the late stage involving macrophage polarization and proliferation, and the regulation of cytokine production. Therefore, we strongly believe that exploring the mechanism of immune cells and chemokines in future research will promote the rapid development of targeted therapy for IHD.

### 3.3 Diverse regulation of macrophage polarization

As innate immune cells, macrophages are extremely phenotypically plastic and exhibit specific functional phenotypes regulated by different environmental cytokines, which are responsible for their various roles in host defence, wound healing, and tissue homeostasis dynamics. In the initial stages of IHD, macrophages transition to the pro‐inflammatory M1 subtype. This transformation is crucial for the removal of apoptotic and necrotic debris, facilitating the migration and proliferation of fibroblasts and endothelial cells, and for preparing the groundwork for subsequent fibrotic repair processes.^27^, ^47^ On the 4–7 days after IHD, these macrophages develop the M2 phenotype, which is characterized by the secretion of numerous growth factors that are vital for vascular regeneration and cardiac healing. However, an overly robust inflammatory response can lead to matrix degradation, increase the risk of cardiac rupture, and impair collagen deposition, ultimately contributing to ventricular dilation.^27^, ^48^ A deep understanding of the mechanisms governing macrophage polarization is crucial for enhancing the inflammatory response and promoting repair mechanisms following IHD. It is widely believed that macrophage polarization is influenced by various factors in the tissue microenvironment, including metabolic changes in macrophages, cytokines, growth factors, and molecular patterns associated with microorganisms. These signals drive the transcriptional response that ultimately defines the phenotypic and functional traits of macrophages in both physiological and pathological conditions.^9^

Metabolism is the basis of all biological functions, and organisms need different environments to achieve the rational allocation of limited resources to grow and reproduce in specific environments. Similarly, the metabolic pathways of immune cells also change in response to changes in the microenvironment, and changes in metabolic pathways also lead to changes in immune cell function, which is called metabolic reprogramming.^49^, ^50^, ^51^, ^52^ The polarization of macrophages may be closely related to their metabolic reprogramming. Glucose metabolism is the main method of cellular energy synthesis and is the basis for maintaining the life activities of organisms. Activated macrophages obtain energy through different glucose metabolism pathways. M1 macrophages mainly rely on the glycolytic pathway for energy to clear invading pathogens, whereas M2 macrophages rely on the oxidative phosphorylation of the tricarboxylic acid cycle for energy.^50^, ^53^, ^54^ Thus, factors affecting macrophage metabolism, such as increased production of inflammatory necrotic products, macrophage pressure overload products, macrophage pressure overload, and cellular ROS generation factors affecting macrophage metabolism, disrupt the M1/M2 balance, accelerating the deterioration of post‐infarction inflammation and leading to severe adverse ventricular remodelling.^51^

Furthermore, macrophage polarization is influenced by signals from the tissue microenvironment. In the context of IHD, cardiac tissue contains a high concentration of early M1 macrophage activation factors, including numerous chemokines and inflammatory cytokines generated as a result of ischemia and oxidative stress, such as TNF‐α and IFN.

#### 3.3.1 TNF‐α

TNF‐α is a highly efficient inducer of inflammatory responses and plays a key role in activating neutrophils and mediating inflammatory responses. The release of large amounts of TNF‐α after myocardial infarction promotes inflammatory injury, inducing the synthesis of chemokines and adhesion factors in the infarcted myocardium.^55^, ^56^ In in vitro experiments, continuous intracellular initiation of cascade responses involving NADPH oxidase, Nox2, and Nox4 and the eventual production of pro‐inflammatory cytokines such as IL‐1β and IL‐6 were detected when cardiomyocytes were stimulated with TNF‐α.^57^ IL‐1β also significantly induces and mediates inflammatory leukocyte recruitment and activation in infarcts while delaying myofibroblast activation.^58^ IL‐6 is also upregulated in infarcted hearts and may regulate inflammatory and repair response signalling via the IL‐6 receptor subunit β and activation of the Janus tyrosine kinase/signal transducer and activator of transcription (JAK/STAT) pathway.^59^ These cytokines can direct inflammatory cells to the damaged myocardium and produce further inflammatory mediators, thereby establishing an inflammatory response. Studies on TNF‐α knockout mice revealed that inhibition of TNF‐α production attenuated the inflammatory response after myocardial injury, leading to a reduction in reparative myocardial fibrosis and improvement in cardiac function.

In addition, TNF‐α stimulation results in the formation of polymorphonuclear neutrophils (PMNs), and exosomes (TNF‐Exos) isolated from their supernatants promote M1‐type macrophage polarization in vivo and in vitro, further promoting the secretion of cytokines.^60^ The miRNA analysis of TNF‐Exos produced by PMNs revealed that miR‐30d‐5p expression was significantly greater in TNF‐Exos than in PBS‐Exos. More importantly, miR‐30d‐5p levels decreased in TNF‐α‐stimulated PMNs, indicating that miR‐30d‐5p was transferred from within the cells to the exosomes, whereas macrophages incubated in vitro with TNF‐Exos exhibited increased levels of miR‐30d‐5p. This finding suggested that TNF‐α enhances the loading of miR‐30d‐5p from PMNs to exosomes and the transfer of miR‐30d‐5p to recipient macrophages. Furthermore, recent studies have shown that PMN exosomal miR‐30d‐5p activates the NF‐κB signalling pathway by targeting sirtuin 1, induces M1 macrophage polarization, and triggers macrophage scorching.^60^, ^61^ These results indicate that TNF‐α plays an essential role in the induction of M1 macrophage polarization in response to inflammation and is an early regulator of the immune response.

#### 3.3.2 Type I interferons

Type I interferons (IFNs) are polypeptides secreted by infected cells that have a variety of physiological functions. First, they induce an inherent antimicrobial state in infected cells and neighbouring cells, thereby limiting the spread of infectious agents. Second, they regulate innate immune responses, promote antigen presentation and NK cell function, and inhibit pro‐inflammatory pathways and cytokine production. Finally, they activate the adaptive immune system, which in turn promotes high‐affinity antigen‐specific T‐cell and B‐cell responses.^62^ Type I IFN is protective against acute viral infections but may have protective or harmful effects on bacterial infections and autoimmune diseases.^62^, ^63^

The type I interferon (IFN) response is an innate immune pathway known for its role in antiviral defence and link to autoimmune conditions like lupus.^64^, ^65^ Recent research indicates that aberrant type I IFN responses also occur following heart damage from lack of oxygen.^66^, ^67^ A heart attack leads to the simultaneous demise of numerous heart cells and the widespread release of cell fragments that trigger an innate immune reaction. The death of cells due to lack of oxygen and the phagocytosis of dead cell remnants by activated macrophages stimulate the type I interferon response pathway, contributing to the progression of a heart attack.^67^

Type I IFN, IFN‐α, and IFN‐β are major cytokines involved in the host immune response to viruses and other intracellular pathogens. Activation of the type I interferon response pathway is induced by pattern recognition receptors of the innate immune system, which recognize danger signals from pathogen‐associated molecular patterns.^64^ Type I IFN acts in an autocrine and paracrine manner by binding to the IFN‐α receptor (IFNAR), a cell surface receptor composed of two subunits, IFNAR1 and IFNAR2.^62^ Inappropriate activation of type I IFN can be harmful to the host by promoting an autoinflammatory response and disrupting immune tolerance, leading to autoimmunity.^64^

Animal experiments have shown that inhibiting IFN signalling by drug or genetic means can reduce inflammation, limit poor ventricular remodelling, and improve survival in mice.^65^ We suggest that temporary suppression of interferon‐dependent innate immune responses to ischemic cell death due to myocardial infarction can reduce post‐infarction inflammation and effectively limit adverse ventricular remodelling that leads to severe heart failure. Human‐tolerated anti‐interferon biologics are being developed for clinical research on rheumatism, which also indicates the feasibility of targeting the interferon response for AMI in the future.^68^, ^69^

#### 3.3.3 IRF5

IRF5 is a member of the interferon regulatory factor (IRF) family with various biological activities, such as the activation of genes encoding type I interferons, inflammatory factors (TNF, IL‐6, IL‐12, and IL‐23), and tumour suppressors.^27^, ^70^ In response to inflammatory or dangerous stimuli, IRF5 induces the expression of inflammatory genes, leading to the polarization of M1 macrophages. M1 macrophages are characterized by a large upregulation of IRF5 expression, and the upregulation of M1 macrophage‐related genes leads to the persistence of inflammatory M1 macrophages, which affects the healing process and impairs organ function.^71^, ^72^

IRF5 regulates macrophage function and phenotype, facilitating inflammatory responses and macrophage polarization.^73^ Elevated IRF5 levels were observed in macrophages within mouse myocardial tissue compared to normal myocardial tissue on day 4 of peak post‐AMI inflammation. The substantial reduction in IRF5 levels in macrophages by day eight post‐infarction aligns with the shift from M1 to M2 macrophages around days 5 to 7 post‐infarction. Prolonged inflammatory monocyte and macrophage activity following ischemic injury hinders infarcted myocardium healing, potentially leading to heart failure.^74^

IRF5 regulates a series of pro‐inflammatory genes by controlling the expression of downstream pro‐inflammatory cytokines involved in MyD88‐dependent Toll‐like receptor signalling, which exacerbates the inflammatory response after myocardial infarction.^75^ IRF5 can directly regulate the expression of TNF‐α and IL‐1β.^71^ In a mouse model of myocardial infarction, knockdown of IRF5 alters macrophage polarization, accelerates inflammatory regression, increases fibrosis in the infarct zone, accelerates the rate of tissue repair and attenuates heart failure.^74^, ^75^ Thus, IRF5‐regulated genes may critically influence the inhibition of infarct tissue healing and the improvement of post‐infarct heart failure and poor ventricular remodelling.

Various studies have shown that the role of IRF5 in IHD is closely related to that of macrophages.^73^ Inhibition of IRF5 expression in vivo reduced the expression of M1 macrophage‐specific genes without affecting the expression of M2 macrophage‐specific genes. Therefore, in the future, IRF5 could be considered a new target for interventional therapy after the acute inflammatory response after AMI. Inhibition of IRF5 activity effectively reduces the expression of pro‐inflammatory cytokines, attenuates the inflammatory response in post‐infarcted tissues, and promotes healing and tissue remodelling of the infarcted myocardium, which may make IRF5 a potential therapeutic target for the treatment of IHD.^71^

### 3.4 Macrophage‐induced fibroblast activation promotes infarcted myocardial repair

Following acute myocardial infarction, the myocardium undergoes a complex repair process that unfolds in three sequential stages: inflammation, proliferation, and maturation. Initially, during the inflammation stage, the extensive death of heart muscle cells in the affected area triggers the body's innate immune defences, leading to a robust inflammatory reaction. This response is crucial for clearing dead cells and the remnants of the extracellular matrix within the damaged zone. Subsequently, in the proliferation stage, the surviving cardiomyocytes begin to produce collagen, a key step that aids in the healing of the infarcted tissue. Moreover, monocytes and macrophages contribute to this process by releasing growth factors that stimulate fibrotic repair mechanisms. When myocardial tissue is ischemic or necrotic after IHD, the dead cells in the myocardium are replaced by fibrotic tissue. Myocardial fibrosis leads to scarring, which protects heart muscle to a certain extent.^27^ Finally, in the maturation phase, inflammatory activity significantly decreased, indicating that the repair process was nearly complete. At this point, the myocardial tissue has largely recovered, with the post‐infarct area having undergone substantial repair and scar formation.^76^, ^77^

Numerous studies have demonstrated that following AMI, TGF‐β produced by macrophages directly induces the expression of pro‐fibrotic genes in fibroblasts, highlighting macrophages and fibroblasts as key contributors to the angiogenic response post MI.^78^, ^79^, ^80^ TGF‐β is a major fibrotic cytokine that influences myocardial fibrosis and LV remodelling. TGF‐β is primarily derived from innate immune cells and is widely present in cardiac myocytes and cardiac fibroblasts. TGF‐β levels in the heart are high during cardiac development and pathology,^81^, ^82^ and TGF‐β is a central mediator of fibroblast activation.

Macrophage‐induced activation of the TGF‐β signalling cascade is a critical bridge between inflammatory and repair responses. In the steady state, TGF‐β is stored in cardiomyocytes and can be rapidly activated after AMI. The production of ROS and the activation of fibrinase result in the activation of TGF‐β in the infarct zone^83^, ^84^; in addition, leukocytes, platelets, and fibroblasts that infiltrate the infarcted myocardial tissue after AMI synthesize and release new TGF‐β, which enhances fibrotic repair.^85^

**FIGURE 4:**
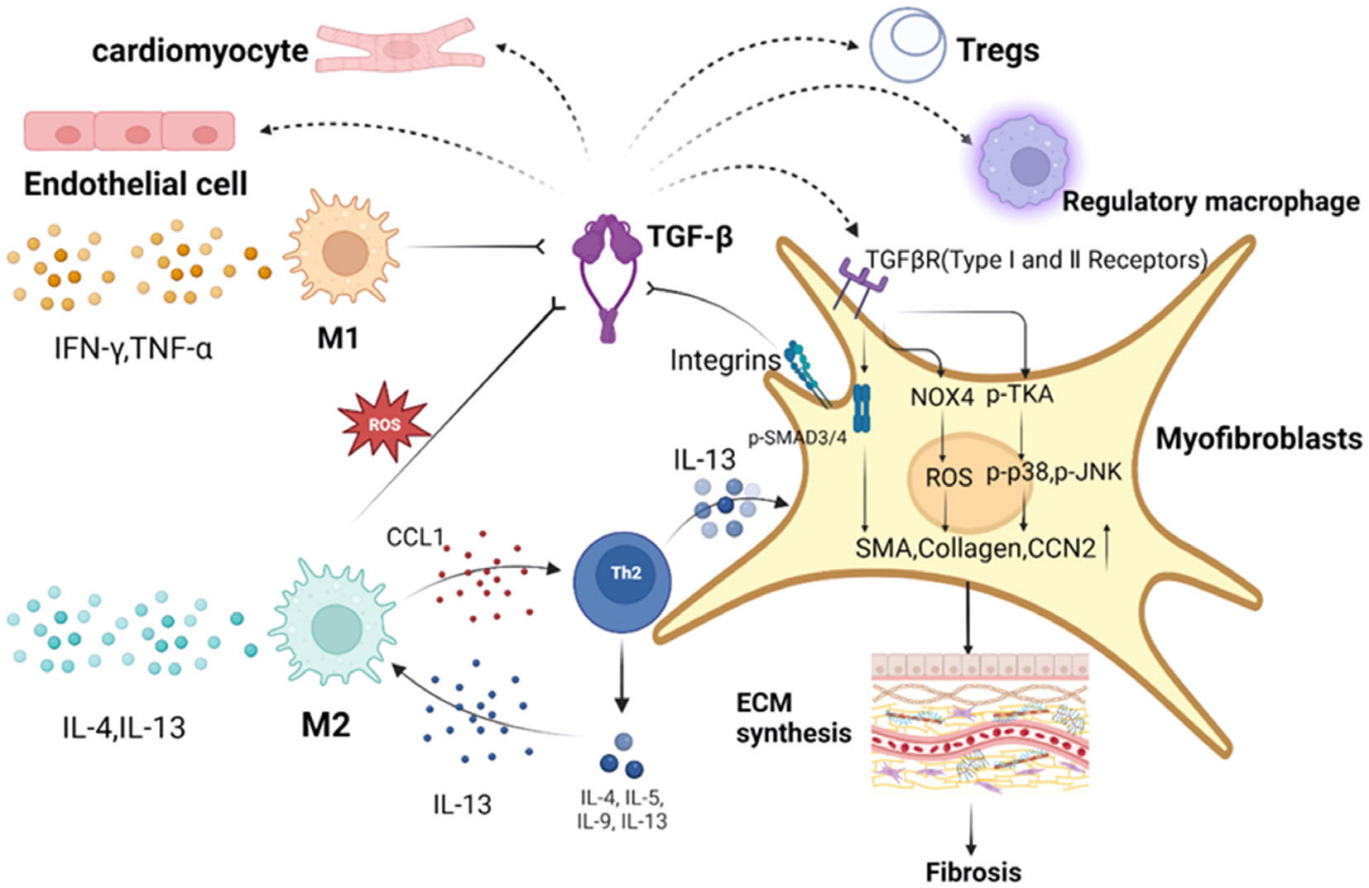
TGF‐β signalling cascade response mediates fibroblast activation after AMI to induce fibrosis. TGF‐β is a major cytokine regulating fibrosis, and its release is regulated by both M1/M2 macrophages. TGF‐β has regulatory effects on endothelial cells, cardiomyocytes, Tregs, regulatory macrophages, and fibroblasts. TGF‐β binds to its type I and type II receptors through integrin‐mediated contractile release, activating the typical Smad3/4 pathway and the atypical TGF‐β‐activated kinase‐1 (TAK‐1)‐p38/c‐Jun N‐terminal kinase (JNK) and NADPH oxidase 4 (NOX4)/ROS pathways, which induce fibrotic genes such as α smooth muscle actin (α‐SMA), collagen and CCN2, to promote extracellular matrix synthesis.

The typical TGF‐β pathway is involved in the development of myocardial fibrosis. The activated TGF‐β/Smad3 pathway mediates myocardial remodelling in the marginal region of myocardial infarct healing.^86^ TGF‐β stimulates cardiac fibroblast migration and induces cell surface integrin expression. TGF‐β binds to its type I and type II receptors via integrin‐mediated contractile release, activating the typical Smad3/4 pathway and the atypical TGF‐β‐activated kinase‐1 (TAK‐1)‐p38/c‐Jun N‐terminal kinase (JNK) and NADPH oxidase 4 (NOX4)/ROS pathways, thereby inducing the expression of fibrotic genes encoding α‐SMA, collagen, and CCN2 and promoting extracellular matrix synthesis (Figure 4). At least some myofibroblasts maintain their fibrotic phenotype via this pathway.^80^, ^87^

Moreover, existing research has shown that guiding the polarization of macrophages toward the M2 phenotype can mitigate excessive fibrosis following myocardial infarction, avert heart rupture, and enhance the repair of cardiac function.^88^, ^89^ This evidence underscores that the involvement of macrophages in the reparative process post‐IHD is not indiscriminate but rather operates within a dynamic equilibrium. Through this balanced action, macrophages not only facilitate fibrotic repair by releasing cytokines such as TGF‐β but also prevent undue fibrosis by undergoing phenotypic shifts, thereby safeguarding cardiac health.

Myocardial infarction triggers a strong inflammatory reaction, which is necessary for heart repair, but excessive inflammation increases the burden on the myocardium and leads to remodelling of the unconsciousness chamber. Timely inhibition of inflammatory reactions is the key to effective healing, after which fibroblasts are activated to secrete matrix proteins in the infarct area. We believe that in the process of treating fibrosis after myocardial infarction, members of the transforming growth factor β family play a key role in inhibiting inflammation and activating pro‐fibrosis procedures. Individuals with hyperactive and prolonged inflammatory responses after infarction may exhibit left ventricular dilatation and contraction dysfunction, and they may benefit from chemokine therapy. Therefore, it is necessary to identify patients with different pathophysiological reactions based on biomarkers and implement appropriate anti‐inflammatory strategies.

## 4 GATA6^+^ PERICARDIAL CAVITY MACROPHAGES

In mammals, the pleural, peritoneal, and pericardial cavities are filled with a significant number of Gata6^+^ macrophages. The role of peritoneal macrophages as phagocytic cells that clear pathogens has been widely recognized. Recent evidence suggests that these cells migrate to injured sites within the peritoneal cavity, where they can promote tissue repair.^90^ It is worth contemplating whether Gata6^+^ pericardial cavity macrophages (GPCMs) have a similar effect, migrating to damaged myocardial sites and facilitating tissue repair after myocardial infarction.

A recent study reported that GPCMs migrated to the heart after ischemic injury. When these macrophages invade the injured heart muscle, the expression of Gata6 (GATA binding protein 6) is lost, but Gata6 can continue to function in repair after myocardial infarction.^90^, ^91^ GPCMs are not limited to mice but are also found in human and pig pericardial fluid and are even more abundant than in rodents, which suggests that GPCMs are a highly conserved group of pericardial immune cells across mammalian species.^90^ Therefore, further study of GPCMs may play an important role in the treatment and repair of myocardial infarction.

GPCMs aggregate in the heart after AMI. Experimental studies have shown the rapid disappearance of GPCMs from pericardial lavage fluid shortly after coronary artery ligation, from day 3 to day 7, followed by a slow rebound in GPCM numbers 28 days after myocardial infarction. A reduction in the number of GPCMs was accompanied by an influx of neutrophils and monocytes into the pericardium, which returned to initial levels 28 days after AMI. During myocardial infarction, GPCMs are recruited from the pericardial cavity to the heart, after which they begin to infiltrate damaged myocardial tissue.^90^, ^91^

We found that GPCMs play important roles in preventing healthy myocardial fibrosis and improving the recovery of cardiac function after ischemic injury. Functionally, when the pericardial cavity is intact, the expression of Gata6‐binding proteins in GPCMs decreases after they enter the heart. In contrast to monocyte‐derived macrophages, GPCMs seem to regulate fibrosis in the local environment of the myocardium.^24^, ^26^ GPCMs affect myocardial remodelling at the distal end of the heart. Because of the increased load on the heart after myocardial infarction, this distal remodelling may be a compensatory mechanism that depends to a great extent on the size of the infarction area and many factors caused by the infarction. Although GPCMs can directly regulate myocardial remodelling in the distal region of the heart, when the compensatory mechanism is excessively activated, it leads to cardiac stiffness and diastolic dysfunction, which further increases the burden on the heart.^92^, ^93^

If GPCMs could infiltrate injured myocardium and contribute to repairing myocardial fibrosis, it could lead to significant progress in macrophage biology and cardiac regenerative medicine. Additional studies may reveal the exact mechanisms that drive the activation, movement, and specific roles of these cells in the cardiac healing process. This understanding presents a fresh angle on recovering from IHD, possibly opening doors to innovative treatment approaches that could enhance patient results and better restore cardiac function.

## 5 NEUTROPHILS IN IHD

After myocardial infarction, in addition to the recruitment of monocytes and the repair function of macrophages, the inflammatory environment of the injured heart attracts a large number of neutrophils.^94^, ^95^ Neutrophils are the first innate immune cells that aggregate in ischemic tissue within a few hours after AMI and gradually disappear by the third day.^11^ The blood neutrophil count and neutrophil‐to‐white blood cell ratio are closely associated with CVDs, such as coronary heart disease, massive myocardial infarction, and atherosclerosis. Atherosclerotic plaque rupture can lead to severe blood circulation disorders, causing ischemic necrosis of tissues, which immediately triggers a severe inflammatory response led by neutrophils. Induced by inflammatory mediators and necrotic cellular debris, neutrophils infiltrate the infarcted area within a few hours.^16^, ^96^ Neutrophils play multiple roles in the myocardial injury response. They enhance the effects of deleterious pro‐inflammatory signals, produce excess reactive oxygen species (ROS) at the site of injury, produce inflammatory factors such as cytokines to influence and regulate inflammatory and adaptive immune responses, and have the ability to trigger the release of protein hydrolytic enzymes that remodel the extracellular matrix.^97^, ^98^, ^99^ Neutrophils can also promote cardiac recovery via mechanisms such as the polarization of macrophages into a repair phenotype, highlighting the potential protective role of neutrophils.^16^, ^94^, ^95^ Therefore, targeted modulation of neutrophil subsets may provide therapeutic opportunities for repair after myocardial infarction. Thus, a comprehensive review of the recruitment and involvement of cardiac neutrophils is highly desirable.

### 5.1 Initial response and regulatory processes of neutrophils

At steady state, granulocyte colony‐stimulating factor (G‐CSF), a growth factor produced primarily by immune cells, fibroblasts, endothelial cells, and bone marrow stromal cells, stimulates granulocyte production.^100^ G‐CSF production is downregulated with increasing neutrophil numbers and upregulated with neutrophil apoptosis. Neutrophils are the most abundant white blood cells and are the first immune cells recruited to the ischemic heart after myocardial infarction. In response to local injury or infection, neutrophils accumulate rapidly and adhere to activated endothelial cells, which in turn are transferred to tissues to engulf pathogens, produce antimicrobial proteins, and generate ROS to eliminate microorganisms whenever possible.^101^ Under certain sterile inflammatory conditions, including ischemic injury due to myocardial infarction, pro‐inflammatory signals accelerate neutrophil activation and production, leading to tissue damage.^16^

**FIGURE 5:**
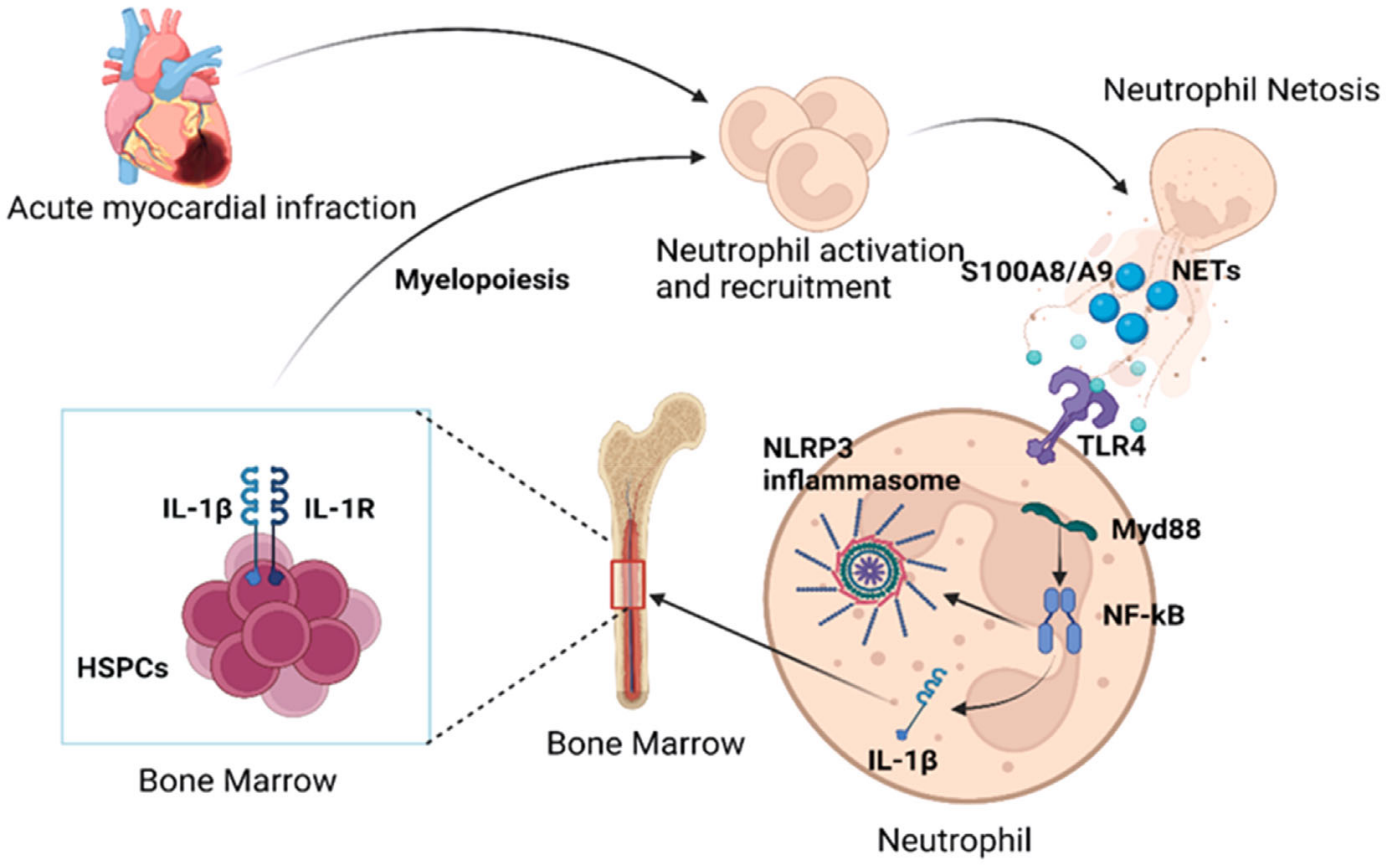
Neutrophil activation and recruitment. During IHD, neutrophils accumulate at the injury site and induce an acute inflammatory response. These neutrophils, through NETosis, release S100A8/A9 into the infarct area, which acts on TLR4 on the cell membrane and causes the release of IL‐1β from the NLRP3 inflammasome on nascent neutrophils, which binds to IL‐1R on HSPCs in the bone marrow, thereby promoting neutrophil recruitment and activation.

Studies have shown that once neutrophils are recruited to the ischemic myocardium, they maintain the initial acute pro‐inflammatory response after myocardial infarction. Their rapid degradation induces an acute inflammatory response to adjacent areas of the myocardium (i.e., neutrophil‐induced injury) and triggers monocyte infiltration into the ischemic region.^102^ For example, the release of S100A8/A9 from neutrophils during AMI can activate NLRP3 inflammatory vesicles, release IL‐1β, and stimulate granule formation in the bone marrow (Figure 5), resulting in neutrophil accumulation and ultimately poor myocardial remodelling and heart failure.^15^, ^16^

However, the release of S100A8/A9 particles after myocardial infarction depends on NETosis.^15^ NETosis is an inflammatory cell death modality. Activated neutrophils capture and kill pathogens by releasing neutrophil extracellular traps (NETs) composed of depolymerized chromatin and intracellular granule proteins to the cell exterior.^103^ NETs consist of double‐stranded DNA that is modified by pro‐inflammatory cytokines and enzymes such as myeloperoxidase (MPO). NETs can bind to endothelial cells (ECs) via vascular hemophilic factor (vWF) and p‐selectin, which provide scaffolding for platelets, erythrocytes, and neutrophils to bind to, leading to fibrin deposition and thrombotic microvascular occlusion.^104^, ^105^, ^106^, ^107^, ^108^ The formation of NETs is accompanied by neutrophil death or NETosis, a novel form of death that differs from apoptosis and necrosis.^109^

### 5.2 Neutrophil function in IHD

Neutrophils, which are traditionally considered the immune system's first line of defence, have gained recognition for their crucial role in sterile inflammation, especially in the context of heart damage in individuals with IHD. In addition to eliminating only dead cells and bacteria, these cells are instrumental in post‐myocardial infarction repair through their robust phagocytic capabilities.^45^, ^110^, ^111^ Under conditions of ischemia and hypoxia, neutrophils generate ROS, NETs, and a variety of inflammatory mediators. These substances not only contribute to initial inflammatory damage but also play a vital role in activating and directing the subsequent immune response.^112^, ^113^ Although it is usually considered harmful in the case of heart attack, recent studies have revealed the anti‐inflammatory and tissue repair effects that neutrophils can have during IHD. The macrophages in the ischemic myocardium are highly plastic, transition from M1 to M2, and participate in tissue injury and repair processes.^45^ Neutrophils are proven to crucially aid the transformation of monocytes and macrophages.^111^ Therefore, we focused on the key role of neutrophils in mediating the inflammatory response and myocardial repair after ischemic heart disease, providing new ideas for the treatment of IHD via immune cells.

#### 5.2.1 Neutrophils and inflammation in IHD

Mammalian neutrophils are important contributors to infection and sterile inflammatory immune responses.^114^, ^115^ In recent years, the role of neutrophils in aseptic inflammation associated with CVD has received extensive attention through in‐depth studies of the inflammatory mediators released by neutrophils. We have noted that neutrophils may play roles in the inflammatory response beyond our traditional understanding.

Infiltrated neutrophils release a large amount of MPO in inflammatory reactions.^116^ Although inflammation is very important for heart healing after myocardial infarction, excessive inflammation induces the release of large quantities of neutrophils, which leads to poor cardiac remodelling and an increased risk of secondary adverse vascular events.^117^ MPO is a heme enzyme that is abundantly expressed and released in neutrophils and monocytes and is a key mediator of inflammation. MPO exhibits cytotoxicity by generating reactive oxygen species, such as hypochlorous acid, in the presence of its substrate, hydrogen peroxide (H_2_O_2_).^118^ MPO can also play a key role in the regulation of vascular function by limiting the bioavailability of nitric oxide (NO), exacerbating endothelial dysfunction and cardiovascular disease development.^119^, ^120^ In addition, plasma MPO levels are strongly associated with the prognosis and severity of myocardial infarction and are important biomarkers for identifying high‐risk patients.^121^, ^122^ MPO also has many properties associated with cardiovascular disease, such as effects on apoptosis, NETosis, neutrophil migration and activation, platelet function, adaptive immunity, endothelial dysfunction, vascular function and leukocyte recruitment.^123^, ^124^ Overactivation of neutrophils leads to increased cardiac damage, whereas complete depletion of the PMN leads to uncontrolled fibrosis and decreased cardiac function after myocardial infarction.^111^, ^116^

Ischemic heart disease is a multifaceted inflammatory process involving immune cells and inflammatory mediators. Neutrophils and monocytes infiltrate cardiac tissue within the first few hours after injury and play a crucial role in the early stages of inflammation. Neutrophils release large amounts of MPO, which has immunomodulatory effects.^116^ Therapeutic inhibition of MPO has been shown to have a protective effect on cardiac function in mice with myocardial infarction by reducing neutrophil and monocyte infiltration.^120^, ^122^ Currently, a specific MPO inhibitor is under clinical investigation, which emphasizes the need to advance anti‐inflammatory therapy after myocardial infarction.^125^, ^126^ Moreover, we found that MPO‐induced chemokine receptor expression is the driving mechanism for increased cardiac monocyte infiltration after myocardial infarction; therefore, we believe that dual therapy using MPO and chemokine receptor inhibition can be considered an anti‐inflammatory treatment for ischemic heart disease.

Massive cardiomyocyte death after IHD effectively activates the innate immune response. The subsequent infiltration of neutrophils, monocytes, macrophages, dendritic cells and lymphocytes into the ischemic region promotes inflammation and regression; the clearance of cellular debris; angiogenesis; and ventricular remodelling, which ultimately determines the degree of deterioration of cardiac function.^127^ A growing body of experimental data suggests that the subtle changes in the ability of neutrophils to mediate beneficial and detrimental aspects of their inflammatory and regulatory capacities deserve further investigation. Neutrophils have long been suspected to play a destructive role in the necrotic myocardium after IHD due to their ability to induce oxidative stress in myocardial tissue,^128^ a role that can be attenuated by the inhibition of oxidative enzymes, which in turn modulates the inflammatory response and promotes the recovery of cardiac function.^129^ However, complete neutrophil depletion unexpectedly leads to increased post‐infarction scar formation and poorer LV function, possibly due to the inhibition of repair functions.^111^ When MPO activity was inhibited, neutrophil infiltration was reduced, and the post‐infarction inflammatory response was attenuated; therefore, the use of a therapeutic MPO inhibitor might provide a new means of treating IHD and facilitating the transition from experimentation to clinical practice as soon as possible. In addition, neutrophils play both a damaging role and a repairing role in the cardiovascular inflammatory response. The dichotomy of neutrophil activity deserves to be explored in greater depth, and its function may change in response to environmental changes. In any case, the dichotomous function of neutrophils is worth considering when designing therapeutic strategies for neutrophil‐targeted treatment of cardiovascular inflammation.

#### 5.2.2 Neutrophils and myocardial repair

**FIGURE 6:**
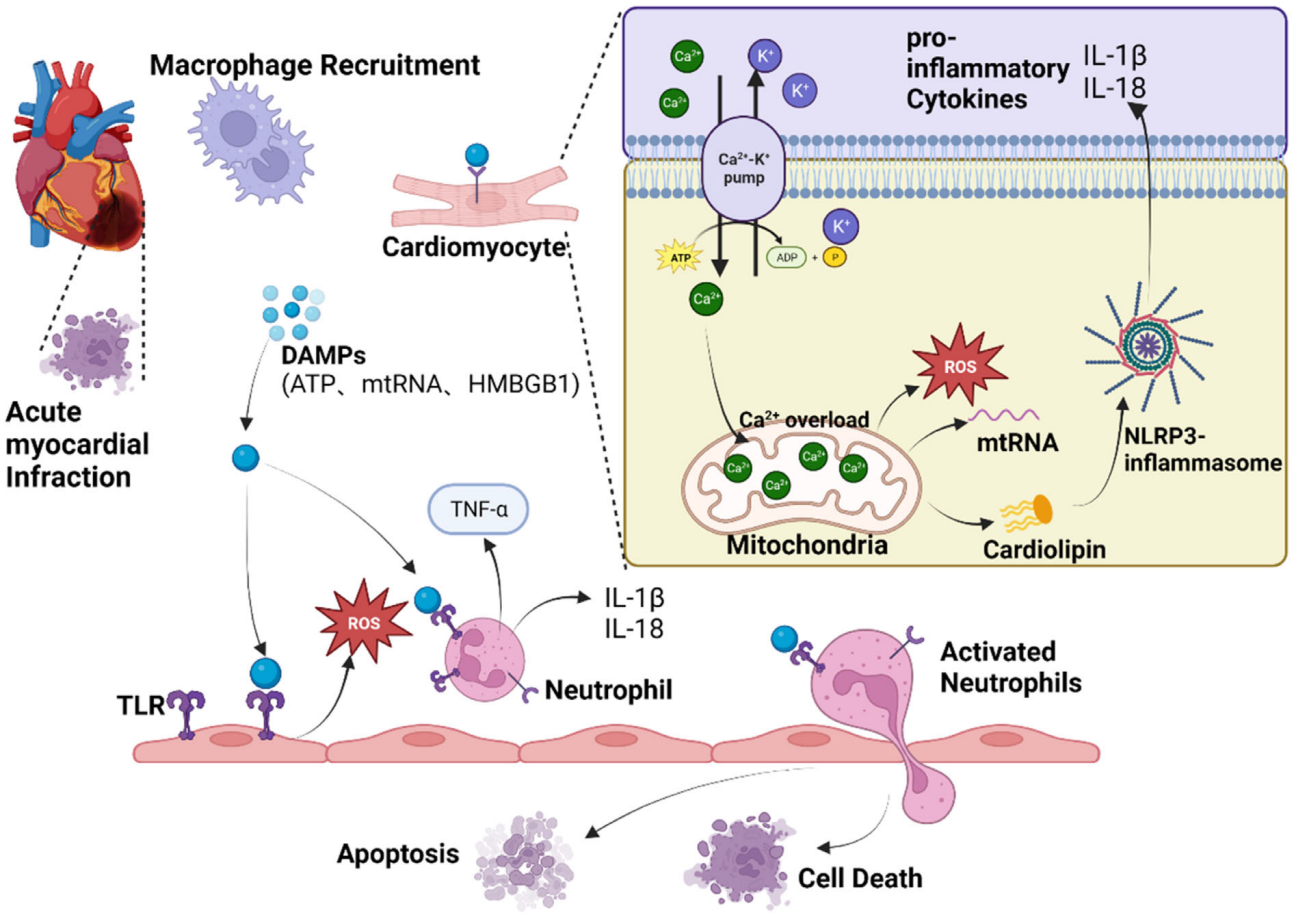
Activation of inflammatory vesicles by neutrophils after AMI induces cell necrosis and apoptosis. After AMI, inflammatory tissue‐recruited macrophages release DAMPs (ATP, mtRNA, RNA and HMBGB1) to exacerbate the inflammatory response, and these inflammatory signalling factors mediate cardiomyocyte death by acting on TLRs, causing intracellular calcium overload and ROS production, promoting RLRP3 inflammatory vesicle formation, or bind to neutrophil surface receptors, causing neutrophil activation, inducing a sustained inflammatory response, which in turn leads to apoptotic cellular tissue necrosis.

During AMI and ischemia/reperfusion‐mediated injury, neutrophils participate in myocardial repair and remodelling responses, in addition to their known pro‐inflammatory effects. After neutrophil infiltration to the site of injury, monocytes and macrophages recruited by monocytes aggregate in the infarcted area, remove apoptotic neutrophils and necrotic cell debris, and release pro‐inflammatory inhibitory factors such as IL‐10 and TGF‐β to activate the initiation of normal tissue repair responses.^5^ The release of S100A8/A9 by neutrophils induces the expression of Nr4a1 and promotes the conversion of inflammatory macrophages to reparative macrophages (Figure 6).

Neutrophils play a vital role in cardiac remodelling and repair following myocardial infarction and are crucial initiators of the inflammatory response that coordinates the removal of necrotic tissue. They are among the first leukocytes to be drawn to the infarcted left ventricle. Neutrophils generate various inflammatory mediators (e.g., cytokines and chemokines), and when activated by ROS, they release granule components (e.g., proteases) to manage the wound healing process through degranulation.^130^ Following IHD, infiltrating neutrophils degranulate and attract more immune cells, thus intensifying the inflammatory response. Alterations in the inflammatory environment influence macrophage programming and adjust their various functions. Neutrophils in the infarcted region also modify their phenotype.^130^

Similar to macrophages, neutrophils are generally categorized as N1 or N2 cells. On days 1–3 of acute inflammation, N1 cells express pro‐inflammatory markers such as IL‐1β, TNF‐α, and S100A8/A9, indicating that the pro‐inflammatory cytokines they release promote M1‐like polarization of macrophages. From days 4–7 of inflammation, N2 cells increase the expression of anti‐inflammatory markers such as CD206, TGF‐β, and IL‐10, suggesting that N2 cells release anti‐inflammatory cytokines that promote M2‐like polarization of macrophages. This provides strong evidence that neutrophils play a role in influencing the polarization of monocytes and macrophages. Hence, it is likely that pro‐inflammatory N1 neutrophils play a role in initiating M1 macrophage polarization through the secretion of TNF‐α and INF‐γ in the inflammatory phase following IHD. In contrast, anti‐inflammatory N2 neutrophils might contribute to promoting macrophage M2 polarization in the post‐inflammatory reparative phase.^131^, ^132^

In addition, neutrophils can produce membrane‐linked protein A (Annexin A), which activates the angiogenic phenotype of macrophages and promotes vascular regenerative repair via an indirect mechanism.^96^, ^130^, ^133^ Neutrophils store Annexin A1 in their granules, and during inflammation, newly synthesized Annexin A1 is released into the microenvironment as microcystic contents.^134^ Phosphorylated Annexin A1 binds to the G‐protein‐coupled receptor formyl peptide receptor (FPR) in a calcium‐dependent manner,^135^ exerting autocrine and paracrine effects to halt inflammation.^136^ Moreover, decreased Annexin A expression at the injury site in neutrophils exacerbates and prolongs inflammation in inflammatory diseases.^137^

Previous studies have shown that melanoma cells in the microenvironment express Annexin A1, which is associated with angiogenesis, tumour cell invasion, and growth.^138^, ^139^ Therefore, we examined the potential role of Annexin A1 in the inflammatory environment following IHD. Exploring the participation of Annexin A1 in neutrophil‐mediated activation could be vital for future research on post‐infarction myocardial repair and angiogenesis.

Animal studies have shown that prolonged neutrophil depletion in myocardial infarction caused by permanent left anterior descending coronary artery ligation results in dysfunction of the cardiovascular system, increased myocardial fibrosis, poor ventricular remodelling, and ultimately heart failure.^111^ Short‐term inhibition of S100A8/A9 production by neutrophils, a significant reduction in the myocardial infarct area, and an increase in the LVEF were found upon administration of intravenous metoprolol (a β‐blocker) early in myocardial ischemia–reperfusion without significant adverse events within 24 h. In contrast to short‐term inhibition, long‐term inhibition of S100A8/A9 resulted in sustained deterioration of cardiac function and irreversible damage. Therefore, clinical treatment at an appropriate therapeutic window should be selected to effectively inhibit the inflammatory function of neutrophils and prevent them from causing excessive damage to tissues while preserving their repair function.^96^, ^133^ Therefore, interventions that inhibit NETosis, S100A8/A9, and IL‐1β during the acute inflammatory phase after myocardial infarction may exert beneficial effects on the post‐infarction inflammatory process and ventricular remodelling.^15^

Neutrophils play a key role in the inflammatory response during infection and tissue injury and in maintaining homeostasis in vivo. Exploring the balance between pro‐inflammatory N1 and anti‐inflammatory N2 properties in neutrophils for enhanced post‐infarct repair in IHD is a pressing topic in current research, potentially leading to the application of neutrophil biological intelligence in clinical practice. We believe that neutrophils are indispensable and play an important role in the study of immune activation in ischemic heart disease, but at the same time, we should also pay attention to the careful balance of treatment strategies for neutrophil‐driven inflammation, fully grasp the relationships among different subtypes of cells, and minimize the adverse effects on healing reactions and cardiac remodelling.

## 6 DCs IN IHD

Dendritic cells (DCs) are efficient antigen‐presenting cells that regulate immune responses by promoting immunity or establishing immune tolerance, connecting innate immunity to acquired immunity. It is now understood that DCs comprise a diverse subpopulation, each with unique effector functions that influence acute and chronic inflammation.^140^

In the inflammatory response induced by AMI, in addition to the immune cell response described above, DCs also migrate and aggregate toward the ischemic zone. Animal experiments have shown that mice with massive DC depletion have worse post‐infarct ventricular remodelling than control mice, indicating that DCs exert a protective influence on the pro‐inflammatory response in the early phase of myocardial infarction. We also found neutrophil activation, pro‐inflammatory monocyte and macrophage recruitment, and increased pro‐inflammatory cytokine levels in the infarcted myocardium of mice with massive DC depletion. In addition, DC levels decreased, macrophage numbers increased, and fibroblasts were heavily activated during repair, leading to excessive myocardial fibrosis in histological specimens from patients with myocardial infarction‐induced cardiac rupture; this increased the stiffness of the necrotic heart and risk of rupture, indicating a protective function of DCs against cardiac rupture.^20^, ^141^, ^142^

### 6.1 Function of tolerogenic DCs (tDCs) in IHD

In this section, we will discuss a specific type of DC, tolerogenic dendritic cells (tDCs). In different DC subpopulations, tolerogenic dendritic cells (tDCs) play an important role in inducing peripheral tolerance through specific mechanisms, including activation of regulatory T cells (Tregs), inhibition of effector T cells, and negative regulation of Th1/Th2 immune responses. Although the exact mechanism by which tDCs are induced to become immunogenic or tolerogenic in vivo has not been elucidated, there is growing evidence that the function of tDCs is essential for maintaining immune balance.^143^, ^144^ Here, we focused on the activation of Tregs by cardiac‐specific tDCs, followed by the induction of their conversion to a reparative phenotype by promoting macrophage polarization and increasing angiogenesis and tissue repair in the infarcted area to coordinate healing.

Activation of tDCs after myocardial infarction reduced the myocardial infarct size and improved left heart function and survival in mice with myocardial infarction. Twenty‐eight days after myocardial infarction, cardiac magnetic resonance imaging revealed that mice treated with tDCs had significantly reduced left ventricular dilatation, significantly enhanced left ventricular systolic function, and well‐healed trauma in the infarcted area, with significantly greater survival rates than did control mice.^145^ This finding suggested that tDC treatment reduces adverse remodelling in the infarct zone, improves left ventricular systolic function, and does not negatively affect the healing of the infarct zone after trauma.

In infarcted mice, tDCs can induce Treg activation. Injection of tDCs into mice revealed that Treg production in the spleen was significantly greater than that in the control group. Moreover, at 5 and 7 days after infarction, the expression of IL‐4 and IL‐10 increased in the tDC‐injected mice, demonstrating its effect on tissue repair. Moreover, the absolute value of Treg infiltration in the junctional and infarcted areas was significantly greater in tDC‐treated mice than in control mice, and the expression of VEGF in the infarcted area was high in the treatment group, suggesting that the use of tDCs may promote neovascularization in the infarcted area.^141^, ^145^ These results indicated that tDCs increased Treg recruitment in the infarcted myocardium and lymphoid organs and promoted trauma healing in the infarcted myocardium by inducing angiogenesis.

Inducible activation of Tregs by tDCs similarly induces rapid conversion of macrophage subsets in the infarcted myocardium. A recent study showed that the application of tDCs in mice with acute heart infarction induces Treg activation, which in turn affects monocyte recruitment and promotes the conversion of M1‐type macrophages to M2‐type macrophages, which in turn improves cardiac remodelling and function in post‐infarction mice and increases survival. M1 macrophages are the critical components that appear after AMI, although long‐term activation is harmful for body tissues, while M2 macrophages are essential for tissue wound healing because of their timely appearance.^59^, ^145^, ^146^ Tregs play a central regulatory role in coordinated macrophage recruitment and polarization.^147^

The distribution of Tregs and M1 and M2 macrophages in the myocardium was examined by immunofluorescence staining, and the cytokines produced by M1 and M2 macrophages were analysed to assess the dynamics of macrophages after tDC injection. We concluded that the levels of M1‐specific cytokines such as IL‐1β, IL‐6, IL‐12B, and TNF‐α decreased 3 days after tDC treatment, while those of M2‐specific cytokines such as Arg‐1, Mrcl1, and Mgl1 increased significantly by the fifth day of tDC treatment.^145^, ^148^ This discovery suggested that tDCs help regulate the immune response effectively by activating Tregs and swiftly converting M1 macrophages to the M2 phenotype. This process aids in reducing inflammatory damage, enhancing wound healing, and improving ventricular remodelling following AMI.

### 6.2 Perspectives on DCs

Ventricular remodelling after myocardial infarction is an inevitable process that includes changes in the geometry and stiffness of the ventricle, as well as changes in the genetic phenotype and molecular structure of the infarct zone, junctional zone, and distal myocardium. Although advanced clinical measures have been adopted to save the lives of most patients, preventing the occurrence of left ventricular systolic‐diastolic dysfunction and heart failure is difficult.^149^, ^150^, ^151^ Subcutaneous injection of DCs significantly improves left ventricular remodelling and induces a shift from a pro‐inflammatory to an anti‐inflammatory environment as soon as possible after AMI, providing a favourable external environment for myocardial tissue repair and thus improving cardiac function.^145^ In addition, tDCs have been shown to be natural inducers of Tregs in vivo and are involved in the immunomodulatory effects of post‐infarction myocardial inflammation. Activated Tregs participate in the myocardial inflammatory phase transition, controlling the conversion of macrophages from the M1 to M2 phenotype and promoting favourable ventricular remodelling; conversely, depletion of Tregs impairs function in the repair phase, leading to the persistence of inflammatory M1 macrophages and delayed tissue repair in the infarcted area.^145^, ^152^ tDCs induce systemic activation of specific Tregs after AMI, thereby inducing a shift in early macrophage subpopulations from inflammatory M1 to reparative M2 macrophages.^152^ An altered immune environment within the infarcted heart results in better wound remodelling and preservation of left ventricular systolic function after myocardial tissue injury and improves survival.

Several animal experiments have shown that tDC therapy is safe, does not cause autoimmune problems, and has consistently shown promising results in improving poor remodelling and survival after myocardial infarction^153^; however, extensive validation is needed to determine whether these experimental results can be applied in the clinic.^154^ Therefore, the precise administration of tDCs induces the swift activation of immune cells, such as Tregs, which could help reduce inflammatory reactions and enhance ventricular remodelling following IHD. These findings indicate that tDCs have the potential to serve as a novel treatment approach for myocarditis.

## 7 EOS IN IHD

EOSs are usually considered to be toxic effector cells.^155^ EOSs mature in the bone marrow mainly under the regulation of the transcription factors GATA‐1, IL‐3, IL‐5, and GM‐CSF. The cytoplasm of EOSs contains a large number of cationic granule proteins, such as eosinophilic cationic protein (ECP), eosinophilic peroxidase, cytokines (IL‐4, IL‐5, IL‐10, and IL‐13) and chemokines (CCL‐3, CCL‐5, CCL‐11, CCL‐5, and CCL‐11), which are induced when EOSs are activated.^155^, ^156^ EOS is chronically present in peripheral blood and accumulates in damaged areas, particularly in allergic and parasitic diseases where EOS levels are heavily elevated. ECP is a biomarker of EOS activation and is elevated in the plasma of patients with asthma or other inflammatory diseases.^11^, ^155^ However, with advancements in IHD research, the role of EOS in IHD is being gradually revealed.

### 7.1 EOS response in IHD

EOSs are also recruited to the vicinity of the damaged myocardium at the onset of IHD. Changes in the circulating EOS count and eosinophil cationic protein level may be associated with the risk of developing IHD.^157^ Studies have shown that EOS counts in the blood and heart are greater in humans and mice after AMI, especially in the infarcted area, and that congenital eosinopenia or induced eosinophil depletion exacerbates post‐infarction cardiac dysfunction, cell death, and fibrotic processes, accompanied by increased levels of neutrophils, monocytes, and macrophages. Furthermore, EOSs that infiltrate the heart exert a protective effect after IHD.^20^, ^129^, ^158^ The expression of eosinophil‐specific IL‐4 and the cationic protein mEar1 can improve the recovery of cardiac function after MI by blocking hydrogen peroxide‐ and hypoxia‐induced cardiomyocyte death and inhibiting fibroblast activation. However, in rare cases, excessive myocardial infiltration of EOS may lead to severe eosinophilic myocarditis.^159^

### 7.2 Function of EOS in IHD

EOS also plays an important role in the diagnosis and treatment of IHD. In patients with myocardial infarction, the circulating EOS concentration is elevated for at least 5 days, the plasma ECP concentration peaks on days 2–3 after infarction, and an elevated ECP concentration is positively correlated with the risk of developing IHD; in patients undergoing percutaneous coronary intervention, the plasma ECP concentration is significantly associated with poor prognosis.^11^, ^160^ However, additional studies revealed a protective role of EOS in IHD and demonstrated that the EOS count can predict the risk of IHD mortality. In a study on EOS counts and IHD‐associated mortality, a strong association was found between low EOS counts and the development of heart failure, malignant arrhythmias, and subarachnoid haemorrhage.^11^, ^161^ EOS levels increase in peripheral blood and damaged hearts after IHD. EOS hampers matrix degradation, angiogenesis, and vascular and inflammatory cell activation by releasing IL4 and cationic proteins. It also boosts SMC proliferation and M2 macrophage polarization, decreases cardiomyocyte necrosis, and obstructs inflammatory cell adhesion and aggregation.^162^, ^163^

The role of EOS in abdominal aortic aneurysm (AAA) has been increasingly demonstrated. EOS and ECP may play pathogenic roles in AAA in humans and mice.^163^ Eosinophil activity during AAA expansion not only impacts adaptive or innate immunity to the lesion but also may affect other anti‐inflammatory processes.^162^ Further studies have shown that EOSs regulate macrophage polarization, decrease myocardial inflammatory cell responses, inhibit cardiomyocyte apoptosis, control fibroblast activity, and manage the adhesion and infiltration of cardiomyocytes after a heart attack. This is achieved by the production of mEar1, IL4, and other cytokines to control myocardial inflammatory cell adhesion and infiltration.^162^ These results provide a theoretical foundation for the mechanism underlying inflammatory regulation by EOS during repair in IHD. Therefore, in the future, EOS and its related proteins and cytokines should be investigated, which may provide new ideas and approaches for treating CVD in the clinic.

## 8 IMMUNOTHERAPY FOR IHD

In chronic cardiac dysfunction, cardiomyocytes cannot proliferate, and the number of stem cells is limited, resulting in the inability of the heart to regenerate. The most common complications after a primary myocardial infarction are heart failure, recurrent ischemia, and arrhythmias. Left ventricular assist device (LVAD) therapy and heart transplantation are currently the main treatment options for end‐stage patients.

LVAD is a life‐saving strategy for some patients with end‐stage heart failure, and it is used as a bridge to heart transplantation, offering patients the opportunity to extend their life and improve their quality of life. Currently, LVAD technology has successfully improved long‐term survival, and the effectiveness of this life‐saving treatment is widely recognized. However, qualitative research on LVAD, including health‐related quality of life tests and psychological symptoms, still lacks meaningful integration.^164^ Studies have shown that patients who wear LVADs experience unique stresses, including changes in body image, battery and device management, bathing and swimming restrictions, and driving and intimacy restrictions, in addition to emotional distress, adjustment difficulties and even disability after the device is implanted.^165^ As a treatment to save the life and improve the quality of life of patients with end‐stage heart disease, heart transplantation still has serious complications, such as chronic kidney failure and immune rejection, and due to the scarcity of heart donors, the proportion of patients who can undergo heart transplantation is very small.

After myocardial injury, the innate immune system‐induced inflammatory response up‐regulates a series of cellular protective responses, such as monocyte activation, neutrophil‐mediated inflammatory response, and macrophage and lymphocyte subsets promoting healing and restoring immune balance, which allows the heart to adapt to increased stress in the short term (physiological inflammation). However, this inflammatory response can become dysregulated, leading to chronic inflammation and left ventricular dysfunction and remodelling.^166^ Therapeutic strategies aimed at reducing cardiomyocyte death, modulating the immune response, and controlling inflammation are essential for the management of IHD.^167^ Based on the limitations of traditional IHD therapies, immunotherapy can precisely regulate the specific immune processes leading to ischemic heart disease, such as reducing the inflammatory response or regulating immune cell activity, improving the pumping function of the heart and promoting the recovery of cardiac function. Moreover, immunotherapy can reduce the risk of disease recurrence and improve patient prognosis by regulating the balance of the immune system. It also reduces the incidence of adverse emotional reactions in patients.^168^ Therefore, novel immunotherapy regimens based on immune cell regulation, such as canakinumab and CAR‐T‐cell therapy, may be useful for treating ischemic heart disease of different etiologies.

### 8.1 Canakinumab

An analysis of the results of the CANTOS trial published in 2019 suggested that targeted anti‐cytokine therapy with the anti‐IL‐1β monoclonal antibody canakinumab reduces hospitalization and mortality in patients with prior myocardial infarction with or without heart failure through the inhibition of IL‐1β signalling.^169^ The CANTOS trial provides compelling results showing that targeting IL‐1β reduces IHD risk and mortality in patients with CVD. In particular, the most significant reductions in the risk of major cardiovascular events, cardiovascular death, and death from any cause were observed with canakinumab in patients with the most significant reductions in IL‐6 and C‐reactive protein (CRP) levels, suggesting that the benefit is associated with targeting the innate immune IL‐1β‐IL‐6‐CRP pathway.^170^ It reduces recurrent cardiovascular events by specifically inhibiting inflammatory pathways. However, the use of canakinumab did not significantly increase overall cardiovascular mortality. In addition, the side effects of this regimen include leukopenia and a higher rate of fatal infections.^170^ In addition to the IL‐1β‐IL‐6‐CRP axis, four other inflammatory pathways are critical for IHD and can potentially be used as drug targets for CVD therapy. These pathways mainly include chemokines and their receptors, cellular immune metabolism, immune checkpoints, hormones, and lipid mediators.^20^, ^170^ Among the various immunomodulatory therapies currently under clinical investigation, the selective activation and amplification of Tregs using low‐dose IL‐2 stand out. Treg deficiency can exacerbate the inflammatory response following IHD, and Treg supplementation has been shown to protect the myocardium and facilitate post‐inflammatory repair. Recent studies have revealed a dose‐dependent increase in Tregs following low‐dose IL‐2 treatment in patients with stable IHD and acute coronary syndrome.^171^ This suggests that leveraging IL‐2 to activate Tregs holds considerable promise as an innovative immunotherapeutic strategy.

### 8.2 Regulatory T cells and CAR‐T‐cell therapy

Chronic inflammation in heart failure is characterized by enhanced adaptive and innate immune responses, followed by a local and even extended systemic increase in the number of immune cells, such as monocytes, neutrophils, DCs, and CD4^+^ T cells, and the production of a large number of pro‐inflammatory mediators.^5^, ^172^, ^173^, ^174^ In recent years, the role of lymphocytes in the AMI‐induced inflammatory response has been extensively investigated; studies have shown that in the course of IHD, in addition to mononuclear macrophage polarization, neutrophil activation, and DC proliferation, antigen‐specific T cells also expand to varying degrees. These cells are abnormally activated, mediate tissue damage, and lead to unfavourable remodelling.^172^, ^175^

A special type of T lymphocyte—Tregs—are considered to be key coordinators of immune homeostasis and the response to external injury and can downregulate the innate immune response.^176^ In addition, animal experimental studies have demonstrated that Treg deficiency impairs the production of helper T cells after AMI, resulting in impaired tissue healing and thus a poor prognostic outcome.^177^, ^178^

Myocardial infarction is characterized by an increase in type 17 helper T cells (Th17) and a decrease in Tregs, resulting in the overproduction of pro‐inflammatory cytokines such as IL‐17A, IL‐17F, and IL‐22,^179^ and these cytokines are positively correlated with serum creatine kinase isoenzyme (CK‐MB), which is a recognized marker of myocardial infarction.^180^ Tregs are a subpopulation of CD4^+^ T cells that play an important role in maintaining immune homeostasis and regulating the progression of inflammatory diseases.^181^ Studies have identified thymus‐derived natural Tregs as Foxp3 (Forkheadbox P3)^+^ cells that circulate in the blood in small amounts in a stable state and induce Tregs produced in some cases by subpopulations of mature T cells, such as tolerance inductions, that produce large amounts of the cytokine IL‐10.^147^, ^182^ FOXP3 is a hallmark of Tregs,^183^ and FOXP3 deletion may lead to Treg dysfunction.^184^ Tregs regulate the activation and function of a variety of immune cells, including innate and adaptive immune responses.^177^, ^182^ As the immune response in IHD has been studied in detail in recent years, the role of Tregs in repairing necrotic tissue in IHD has been increasingly confirmed.

Animal experiments revealed that mice were induced to have myocardial infarction, which caused a large depletion of Tregs in the heart muscle. In addition, the levels of repair cytokines, such as TGF‐β, produced by macrophages decreased significantly. Therefore, it is reasonable to assume that the loss of Tregs leads to impaired resolution of inflammation after acute myocardial infarction, resulting in the persistence of inflammatory macrophages and delayed healing.^147^ During stress following a heart attack, a significant quantity of cellular waste is quickly discharged into the surrounding area, triggering an inflammatory reaction that speeds up the removal of dead tissue and reestablishes heart balance.^185^, ^186^ Animal studies have shown that Tregs expressing the transcription factor ForkheadboxP3 promote cardiac healing.^185^, ^187^, ^188^ In addition, increased T‐cell signalling was also found in the cardiac draining lymph nodes of patients with MI, and Tregs were found in cardiac biopsies.^188^ Tregs can inhibit inflammatory cell clustering at the site of MI and suppress inflammatory factor expression and fibroblast differentiation. Moreover, Tregs can increase M2 macrophage expression, secrete anti‐inflammatory factors through paracrine actions, and promote cell proliferation to aid in tissue repair.^7^

IL‐2 is crucial for T‐cell growth and indirectly contributes to an anti‐inflammatory environment by enhancing the function of Tregs, which helps maintain immune system balance and tolerance after injury.^189^, ^190^ Tregs are highly responsive to the IL‐2 receptor and indirectly suppress T‐cell‐mediated inflammation.^191^ Tregs expressing Foxp3 play a vital role in immune tolerance to antigens. IL‐2 helps convert CD4^+^ T cells to Tregs, demonstrating its importance in immune modulation.^192^ The interaction between IL‐2 and Tregs emphasizes the equilibrium required for a well‐functioning immune system. This control guarantees a specific response to pathogens while averting autoimmunity and inflammation. The diverse functions of IL‐2 underscore its promise as a therapeutic target.

Reduced Treg numbers post‐myocardial infarction can trigger excessive inflammation in the heart, hindering cardiac repair and functional recovery. In the course of chronic congestive heart failure, Tregs lose FOXP3 expression under inflammatory conditions, acquire a pro‐inflammatory TH1 phenotype, and experience adverse left ventricular remodelling.^175^ In addition, during the pathogenesis of CVD, as atherosclerosis progresses, specific Tregs are impaired in function, lose their immunosuppressive ability, and shift to the Th1/Th17 proinflammatory phenotype.^193^ Decreased or impaired Tregs may result in heightened activity of pro‐inflammatory immune cells (e.g., Th1 and Th17 cells), thus amplifying the inflammatory response within the cardiovascular system. Decreased Treg levels may also facilitate cardiac fibrosis development, particularly in specific cardiomyopathies.^194^ However, oxidative stress can result in Treg dysfunction, particularly in settings linked to CVDs. Elevated cholesterol levels also impact Treg function and longevity.^195^

Previous studies have shown that Tregs are beneficial for regulating post‐infarction wound healing, thereby improving clinical outcomes by reducing the inflammatory response in myocardial tissue. On the other hand, therapeutic activation of Tregs can promote the replacement of dead tissue with stable collagen scars, thereby preventing the expansion and rupture of the left ventricle.^196^ T cells, particularly Tregs, play a pivotal role in the pathophysiology of cardiovascular diseases.^197^ Research has demonstrated that deficiencies in both the quantity and functionality of Tregs are linked to a spectrum of cardiovascular conditions. Moreover, interventions aimed at enhancing the function or number of Tregs have been shown to effectively mitigate disease progression.^195^ Consequently, Tregs are increasingly recognized as promising therapeutic targets. A wealth of experimental and clinical evidence suggests that diminished Treg cell populations and compromised functionality are common features across various cardiovascular diseases.^177^ Although the precise mechanisms through which Treg cell therapy benefits cardiovascular health remain to be fully elucidated, this avenue of research holds significant potential.

With the in‐depth study of the role of T cells in ischemic heart disease, chimeric antigen receptor T (CAR‐T) cell therapy has gradually come to the public eye. CARs are modified receptors that instruct lymphocytes (mostly T cells) to identify and eliminate antigen‐expressing cells. The “antigen binding domain” of CARs is responsible for allowing endogenous activation of T cells and subsequent elimination of target cells.^198^, ^199^ CAR‐T cells are often generated in vitro from patient autologous T cells and transduced with a retrovirus or lentivirus containing DNA or RNA encoding a genetically modified CAR protein. Once successfully changed, T cells can be expanded and infused into patients, the congenital T cells of which have been depleted.^200^ CAR‐T‐cell therapy is an innovative treatment option that has been shown to have significant clinical effects on patients with haematological diseases such as acute granulocytic leukaemia.^201^ Recent studies have shown that CAR‐T‐cell therapy can be used for treating CVD.

A growing body of evidence suggests that the regulation of immune cells may benefit a wide range of CVDs, including cardiac fibrosis, coronary atherosclerosis, and heart failure.^202^, ^203^ It is well known that excessive myocardial fibrosis is responsible for poor ventricular remodelling after AMI and that excessive fibrosis results from fibroblast activation. Clinical applications of CAR‐T‐cell therapy are being developed at an accelerated pace, and the number of potential targets is increasing. Fibroblast activation protein (FAP) is the best marker of fibroblasts, is highly expressed in cancer‐associated fibroblasts and a variety of tumour cells, is involved in extracellular matrix remodelling and promotes the migration of stromal and tumour cells. In addition, the number of FAP‐expressing stromal cells has been shown to be correlated with increased levels of angiogenic factors and an immunosuppressive microenvironment.^204^, ^205^, ^206^, ^207^ In recent years, significant results have also been achieved in facilitating CAR‐T‐cell therapy from hematologic to solid tumours by targeting and regulating FAP.^208^ Therefore, for ischemic heart disease, a typical cardiovascular disease, we considered FAP‐targeted modulation as a strategy for CAR‐T‐cell therapy for myocardial tissue fibrosis after myocardial infarction.

Fibrosis both stiffens the myocardium and negatively affects the health and function of cardiomyocytes.^209^ To treat excessive and undesirable fibrosis, the use of CAR‐T cells against fibrosis may provide new therapeutic options.^210^ In recent years, a strategy has been developed by researchers who used a system of lipid nanoparticles (LNPs) encapsulating modified mRNAs to obtain anti‐fibrotic transient CAR‐T cells^211^ and simultaneously designed modified mRNAs encoding the CAR receptor for the FAP protein (FAPCAR). The FAPCAR is expressed by tissue‐damaged cardiac fibroblasts and, subsequently, is encapsulated in anti‐CD5 antibody‐coated LNPs (CD5/LNP‐FAPCAR).^203^ CD5 is a membrane glycoprotein physiologically expressed by T cells.^203^ Researchers have tested the ability of the CD5/LNP‐FAPCAR system to convert cultured T cells into CAR‐T cells and to eliminate FAP‐expressing target cells in vitro. 48 h after intravenous injection of CD5/LNP‐FAPCAR, the researchers found that the expression of FAPCAR was significantly elevated in the T cells of the treated group. Compared to untreated mice, treated mice showed improvements in cardiac volume, systolic and diastolic function, and left ventricular mass.^211^, ^212^ These positive results encourage the use of biomaterials engineered to design CAR‐T‐cell therapies for in vivo dissemination. In addition, experimental animal studies using CAR‐T cells against fap‐mediated fibrosis directly showed that more than half of the mice had reduced or negligible cardiac fibrosis and maintained normal systolic and diastolic function and that peripheral vasculature was not attenuated due to the inability of perivascular fibroblasts to expose FAP directly to the vascular surface.^203^, ^210^

These results show that CAR‐T cells can effectively treat fibrotic mice with minimal cardiotoxicity and inflammatory reactions. By targeting FAP, CAR‐T cells can be used in the clinical treatment of myocardial fibrosis in patients with ischemic heart disease and improve ventricular remodelling, which will undoubtedly provide more comprehensive understanding and strategies for the treatment of cardiovascular diseases.

However, the long‐term effect of CAR‐T cells on the prognosis of patients with ischemic heart disease and cardiovascular disease has not been well studied. A retrospective cohort study showed that the systolic function of 50% of patients with cardiomyopathy/heart failure recovered completely, 25% of patients recovered partially, and 25% of patients whose systolic function continued to decline.^213^ At the same time, CAR‐T‐cell therapy comes with some risks. The most common adverse reaction is the interaction of CAR‐T cells with other immune cells, resulting in the release of large amounts of pro‐inflammatory cytokines, which may lead to various adverse outcomes, such as cardiogenic shock and multiorgan failure.^214^ Furthermore, CAR‐T‐cell therapy has been linked to severe toxicity and cardiovascular complications, including symptomatic heart failure, ischemic stroke, acute coronary syndrome, and de novo arrhythmias.^215^ Based on the advantages and disadvantages of CAR‐T‐cell therapy, we should look at it from a diversified perspective. With the deepening of immunotherapy research, new therapeutic strategies are emerging, which will certainly provide a safer and more effective means for the application of CAR‐T‐cell therapy for the treatment of CVD.

## 9 CONCLUSIONS AND PERSPECTIVES

Systematic studies of immune cells and their inflammatory response have greatly improved our understanding of the progression and development of ischemic heart disease. This article provides insight into the inflammatory response triggered by immune cells in the context of ischemic heart disease from a macro and micro perspective.

From a macro view, the role of immune cells after ischemic heart disease is mainly reflected in their regulation of the inflammatory response and repair processes in the heart. After myocardial ischemia, damaged tissues release signalling molecules, such as cytokines and chemokines, which attract immune cells to migrate to the damaged area. We discuss the various immune cells that contribute to the post‐infarction inflammatory response and subsequent repair response through cytokines, signalling pathways, and other means, in addition to the potential role of Gata6^+^ pericardial cavity macrophages.

From a micro view, immune cells contribute to the inflammatory response through specific molecular mechanisms and cell–cell interactions. We investigated the early release of inflammatory mediators by neutrophils and monocyte‐derived macrophages to coordinate the post‐infarct inflammatory response and create a pro‐inflammatory environment. Additionally, we examined how various cytokines in the microenvironment influence macrophage polarization, the reparative role of M2 macrophages in the later stages of inflammation, the subtle roles of neutrophils and eosinophils in IHD, and how the activation of tDCs alongside the induction of Tregs supports myocardial repair and mitigates unfavourable ventricular remodelling.

Furthermore, this review underscores the significance of macrophage‐induced fibroblast activation in driving the fibrotic reaction following myocardial infarction. This finding underscores the pivotal role of the TGF‐β signalling pathway in myocardial fibrogenesis, revealing how macrophages prompt the transformation of fibroblasts into myofibroblasts, predominantly via TGF‐β, thereby fostering fibrotic remodelling of infarcted tissue.

Clinical trials focused on immune cells for the treatment of ischemic heart disease are being actively promoted, and methods such as monoclonal antibodies and CAR‐T‐cell therapy are gradually being applied in the clinic. Although we have accumulated much work and experimental data on the role of the immune microenvironment in the repair of heart injury, research on heart repair after ischemic heart disease has several limitations. For example, most treatment methods for heart injury are only used in experimental animals, and the clinical demand for treating heart injury has not been met. Currently, research on ischemic heart injury has focused mainly on ischemia–reperfusion and myocardial infarction, but there are also other types of heart injury, such as heart injury caused by trauma, heredity or viral infection. We believe that in the future, we should strengthen the transformation of existing research into a clinical direction, build more different types of injury models, and expand the curable group of patients with ischemic heart disease through the combination of basic research and clinical application.

In summary, we believe that research on immune response regulation is still the tip of the iceberg. We should fully understand the immune response mechanism in ischemic heart disease and flexibly adjust the balance between activation and inhibition in the future immune response to avoid an excessive inflammatory response. In addition, the emergence of new immunotherapies based on immune cells will certainly provide new ideas for the treatment of cardiovascular diseases. However, due to its limitations, researchers need to deepen their understanding of immunotherapy, broaden their thinking, and apply experimental research to the clinic to treat more patients with cardiovascular diseases. This will be a difficult but worthwhile road.

## AUTHOR CONTRIBUTIONS

All authors have read and approved the final review manuscript. Hongxia Li, Tingbo Jiang, and Tianke Yang contributed to the conceptualization and preparation of the manuscript; Yongjian Zhao, Mingyue Tan, and Yunfei Yin conceived and wrote this review as well as produced the figures; and Hang Li, Yifeng Jin, and Jun Zhang checked the English. Lin Yan, Yiyi Song, and Ziyue Wu reviewed and edited this review; Hongxia Li, Tingbo Jiang, and Tianke Yang supervised this review.

## CONFLICT OF INTEREST STATEMENT

The authors declare that no competing interests exist.
